# A New *In Vivo* Model System to Assess the Toxicity of Semiconductor Nanocrystals

**DOI:** 10.1155/2011/792854

**Published:** 2011-05-05

**Authors:** Angela Tino, Alfredo Ambrosone, Lucia Mattera, Valentina Marchesano, Andrei Susha, Andrey Rogach, Claudia Tortiglione

**Affiliations:** ^1^Institute of Cybernetics “E. Caianiello”, National Research Council of Italy, Via Campi Flegrei, 34, 80078 Pozzuoli, Italy; ^2^Department of Physics and Materials Science, City University of Hong Kong, Hong Kong SAR, Kowloon, China

## Abstract

In the emerging area of nanotechnology, a key issue is related to the potential impacts of the novel nanomaterials on the environment and human health, so that this technology can be used with minimal risk. Specifically designed to combine on a single structure multipurpose tags and properties, smart nanomaterials need a comprehensive characterization of both chemicophysical properties and adequate toxicological evaluation, which is a challenging endeavour; the *in vitro* toxicity assays that are often employed for nanotoxicity assessments do not accurately predict *in vivo* response. To overcome these limitations and to evaluate toxicity characteristics of cadmium telluride quantum dots in relation to surface coatings, we have employed the freshwater polyp *Hydra vulgaris* as a model system. We assessed *in vivo* acute and sublethal toxicity by scoring for alteration of morphological traits, population growth rates, and influence on the regenerative capabilities providing new investigation clues for nanotoxicology purposes.

## 1. Introduction

**Figure 1:**
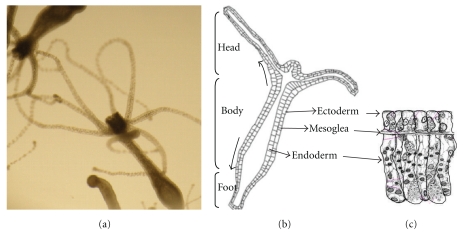
(a) Picture of living *Hydra*. The animal has a simple body plan: it is a tube with a head at the apical end and a foot or basal disc at the other. The head is in two parts, the hypostome (mouth) at the apex and below that the tentacle zone from which a ring of tentacles emerge. Scale bar 200 *μ*m. (b) Schematic representation of the bilayered structure of the animal: the body wall is composed of two self-renewing cell layers, an outer, the ectoderm, and an inner, the endoderm, separated by an extracellular matrix, the mesoglea. The arrows on the left side indicate the direction of tissue displacement. (c) Along the animal body, both ectoderm and endoderm layers are composed of epitheliomuscular cells, while interstitial stem cells and their intermediate and terminal derivatives (neurons, nematocytes, and secretory cells) are interspersed among ectoderm and endoderm.

During the past decade, advances in synthesis and biofunctionalization of colloidal semiconductor nanocrystals have generated an increasing widespread interest among investigators in the field of biology and medicine. The multitude of successful uses of quantum dots (QDs) as specific markers for cellular structures and molecules, monitoring molecular and physiological events in live cells and animals, is a testimony of their great potential as multipurpose bioprobes [1, 2]. However, there exists an open question regarding whether nanoparticles per se can elicit biological responses, which could interfere with the phenomena they are intended to measure. Evidences are cumulating that nanoparticles play active roles even in the absence of specific ligands and that factors such as size and charge are crucial for the activation of cell responses, internalization, and intracellular trafficking [3, 4]. Thus, it is a priority for the wide scientific community working to develop nanostructured materials for biomedical purposes to relate the physical and chemical characteristics of nanomaterials to their behaviour, *in vivo*. While most of the published data addressing this important issue rely on cell culture studies and are focussed on the identification of the physicochemical parameters influencing the impact of nanoparticle on living cells [5, 6], we propose a new model system to work at the whole animal level. The small freshwater polyp *Hydra vulgaris* (Cnidaria, Hydrozoa) is a diploblastic animal, at the base of the metazoan evolution, composed of just two epithelial cell layers (an inner endoderm and an outer ectoderm facing the low ionic strength medium) with few interspersed specialised cell types, a neuronal net controlling functions and physiology (Figure 1). This structural complexity, simpler than vertebrates, with central nervous system and specialized organs, but much complex compared to cultured cells, makes *Hydra* comparable to a living tissue whose cells and distant regions are physiologically connected [7].

The feasibility to approach biological issues using *Hydra* as model system has been shown previously by our group. In a pioneer work, we synthesised glutathione functionalised quantum dots (GSH-QDs), studied the biological activity evoked in living polyps, and identified GSH-targeted cells [9]. In the following studies, we used rod-shaped CdSe/CdS nanocrystals (QRs) not bearing functional groups to identify the mechanisms underlying cell-QR interaction. Unexpectedly, *Hydra* treated with QRs showed a behavioural response, a tentacle writhing activity, which was finely characterized and shown to be calcium dependent and relying on the presence of tentacle neurons. These results indicated that the interactions between living organisms and newly synthesised nanomaterials need to be deeply investigated before employing any new nanostructure for biological purposes, that is, for cell-tracking studies, drug delivery. We have also identified both chemical and biological factors involved in the interaction QR-*Hydra* [4] working both *in vivo*, at the level of whole animal and isolated cells, and *in vitro* on fixed specimens, concluding that the QR internalization is the combined results of QR positive surface charge and membrane trafficking events regulated by the presence of annexin proteins on cell membranes.

A remarkable advantage offered by *Hydra* as a model organism to be targeted by metal-based nanocrystals is the possibility to evaluate the potential toxicity of these nanoparticles on different aspects of *Hydra* physiology. The availability of new animal models suitable for the assessment of nanotoxicity is currently recognised as a priority. *Hydra* is sensitive to a range of pollutants and has been used as a biological indicator of water pollution [10–12]. Metal pollutants such as copper, cadmium, and zinc have been tested against different *Hydra* species, and the relative toxicity based on the median lethal concentration (LC50) for all species was ranked from copper, the most toxic, to cadmium with zinc, the least toxic [13]. Drugs and pharmaceuticals targeted at mammalian receptors have also been shown to adversely affect *Hydra*, showing the feasibility to use this aquatic invertebrate to accurately assess the potential toxicological effect of pharmaceuticals entered into natural waters through sewage effluent and landfill leakages [12]. Several bioassays are available to assess the toxicity of a given compound in terms of acute or sublethal toxicity. Polyps exposure to different drugs may cause (1) alteration of morphological traits and developmental programs, (2) alteration of regeneration or pattern formation; the remarkable regenerative capacity of *Hydra* relies on the presence of mitotically active multipotent stem cells in the gastric region, able to regenerate a new organism within 72 hr; as this process is controlled by temporal, positional, and morphogenetic factors, the presence of toxicants in the medium may affect the full process, and (3) alteration of population growth rates; bioassays measuring *Hydra* population growth by asexual reproduction are rapid, sensitive, and precise. Large numbers of *Hydra* can be cultured due to their small size and rapid reproductive rate [14]. The high reproductive rate of *Hydra* enables subchronic toxicity test which assess the population reproductive effects of a toxicant to be done in short time periods. 

In the present paper, we evaluated the toxicological effects of fluorescent CdTe QDs, presenting different chemical coatings, on a whole organism, *Hydra vulgaris*. By using different approaches, from *in vivo* evaluation of morphological traits to the impact on growth rate and regeneration, we determined different behaviours and toxicological effects played by CdTe QDs, such as the influence of the surface coating, showing the feasibility of using *Hydra* as fast, low-cost, and reliable tool for nanotoxicology studies.

## 2. Methods

### 2.1. Nanocrystals Employed

The water-soluble CdTe QDs used in this study were surface capped with thioglycolic acid (TGA) or glutathione (GSH) and synthesized as described in [15]. In this work, TGA-QDs (mean diameter of 3.1 nm) present an absorption wavelength of the first electronic transition at 537 nm, while GSH-QDs (mean diameter of 3.6 nm) at 598 nm.

### 2.2. Hydra Culture

*Hydra vulgaris* (strain Zurich, originally obtained by P. Tardent) were asexually cultured in physiological solution (SolHy: 1 mM CaCl_2_, 0.1 mM NaHCO_3_, pH 7) by the method of Loomis and Lenhoff with minor modifications [14]. The animals were kept at 18 ± 1°C and fed three times per week with freshly hatched *Artemia salina nauplii*.

### 2.3. In Vivo Experiments with Intact and Regenerating Animals

Groups of 20 animals were collected in plastic multiwells and allowed to equilibrate at room temperature in 300 *μ*L of physiological solution (SolHy: CaCl_2_ 1 mM, NaHCO_3_ 0.1 mM, pH 7). The test was initiated by adding test QDs to each well containing 10 polyps and incubating as necessary. QD uptake was monitored *in vivo*, unless otherwise stated, by continuous video recording using a Camedia digital camera (Olympus) connected to a stereomicroscope (Olympus ZSX-RFL2) equipped with fluorescence filter sets (BP460–490/DM505/LP510). Following extensive washes, *in vivo* imaging was accomplished at several magnifications by using both a stereomicroscope and an inverted microscope (Axiovert 100, Zeiss) equipped with a digital colour camera (Olympus, DP70) and fluorescence filter sets (BP4502013490/FT510/LP515). In order to assay acute toxicity, the morphological changes induced by QD treatment were monitored, by using a scoring procedure of the progressive changes in structure. This procedure allows to examine the ability of animals to recover from QD-induced damage. Every day, using a stereomicroscope, recognizable physical changes in response to different QD ranges were recorded, according to score values (ranging from 1 to 10) described by Wilby [8]. For imaging acquisition and analysis, the software system Cell F (Olympus) was used. For regeneration experiments, treated polyps were bisected in the gastric region and *in vivo* imaged at various time points after amputation. A quantitative method was used for the evaluation of distal regeneration in *Hydra*, based on estimates of tentacle elongation during 14 days of regeneration, determination of a tentacle regeneration index (TRI), and a statistical analysis of profiles obtained from various samples in different experiments [16]. According to this method, for each of the *N* polyps, it is possible to calculate at time *t* the corresponding Tentacle regeneration index (TRI) as follows:

(1) $$R_{j}\left(t\right)=\overset{5}{\underset{K=1}{\sum }}p_{k}\cdot \;\frac{n_{k}^{j}\left(t\right)}{n_{\max\;}}\;j=1,2,...,N,$$

where *n* represents the maximal tentacle number for a single polyp under physiological conditions, that is, *n*_max_ = 8; *n*_*k*_^*j*^(*t*) represents the number of tentacles of class *K* (5 tentacle classes were set, of length equal to 1/8, 1/4, 1/2, 3/4, and1) regenerated by the *j*th *Hydra* at time *t*. The series of TRI values of the *j*th polyp, obtained at the fixed observation times, represents the individual regeneration profile of the polyp. Finally, for each group of *N* = 4  *Hydra*, a mean TRI was calculated at any observation time *t* in order to follow the average regeneration rate of the group. Experiments were performed in air-conditioned environment at 22°C and repeated three times for each condition tested. Median lethal concentrations (LC50) and lethal time (LT50) were calculated using the Spearman-Karber trim method [17].

### 2.4. Hydra Growth Rates

Experimental animals (four *Hydra* with one bud) were treated with the indicated QD, for 4 h, then washed, and the following day placed in 3.5 cm Petri dishes (1 *Hydra*/dish). Control animals at the same developmental stage were not treated. Both experimental and control *Hydra* were fed once daily, and the population doubling time was determined as growth parameter. The growth rate constant (*k*) of an exponentially growing group of animals is defined as ln   (*n*/*n*_0_) = *kt*, where *n* is the number of animals at time *t* and *n*_0_ the number of animals at *t*_0_. For *n*/*n*_0_ = 2, *t* = *T*_2_, the doubling time of the population *T*_2_ was determined by linear regression [18].

### 2.5. Statistical Analysis

LC50 and LT50 values were calculated using the Spearman-Karber trim method [17]. Median scores of morphological condition were compared by nonparametric Friedman analysis [13]. A *t-*test (*P* < .001) was used to test for significance between TRI values within treatments. The slope of the regression curves obtained from single population growth rate was tested for significance using a two-way ANOVA (*P* < .001).

## 3. Results

**Figure 2:**
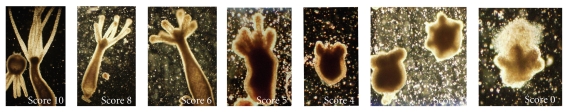
Example of morphological alterations induced by the treatment of living *Hydra* with CdTe QDs. Animals were incubated with increasing doses of TGA- and GSH-capped CdTe QDs, from left to right: 50 nM, 100 nM, 200 nM, 300 nM, 500 nM, 750 nM, and 1 *μ*M over a period of 2 h and then imaged. Progressive morphological changes were scored from 10 down to 1, as previously described in[8].

The two types of highly luminescent CdTe QDs were utilised, thioglycolic acid-capped CdTe QD (from here it is indicated as TGA-QDs) [15] and glutathione-capped QDs (from here named GSH-QDs), and the effects on animal behaviour and morphology where investigated over different incubation times. Being *Hydra* a small water living animal, the simple addition of QDs to the culture medium enables us to study the interaction between QD and animals, avoiding delivering methods or invasive procedures. TGA-QDs and GSH-QDs were added at different concentrations to groups of living polyps which were continuously monitored by fluorescence stereomicroscopy to visually inspect potential QD uptake, localisation, and cell morphology following incubation. By fluorescence microscopy observation, the animals appeared not fluorescently labelled, possibly due to the effect of calcium ions present in *Hydra* culture solution, which have been shown to bleach the QD luminescence [19]. As shown in Figure 2, morphological alterations were induced by the treatment the with both CdTe-based QDs and scored according to previous methods [8]. 

**Figure 3:**
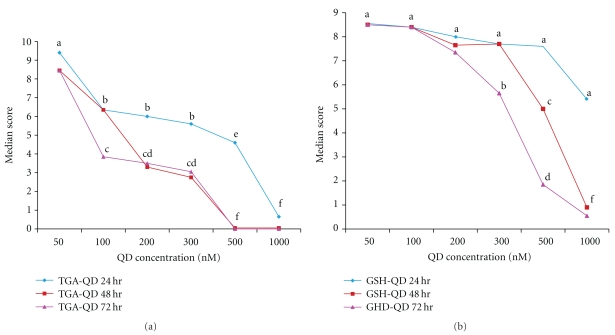
Dose response toxicity curves to (a) TGA- and (b) GSH-capped QDs. 20 *Hydra* were treated with the indicated QD at increasing concentrations for 2 hours and then, following extensive washing, allowed to recover in physiological solution for 24 hr (blue line), 48 hr (red line), and, 72 hr (purple line), when morphology was appropriately scored. Median scores of morphological condition were compared by nonparametric Friedman Test; values with a letter in common are not significantly different (*P* < .001). The toxicity of TGA-and-GSH capped QDs on *Hydra* morphology increases with time and concentration.

A precise and accurate estimation of the median lethal concentration (LC50) was obtained by applying the trimmed Spearman-Karber method, which has good statistical properties, is easy to use, and is recommended for accurate and precise calculation of LC50 values and their 95% confidence interval end points [17]. As this method counts the dead animals and *Hydra* can recover the damage, we considered dead animals as those showing scores lower than 4. Median scores recorded at each QD test concentration of treated animals decreased with increasing exposure, concentration, and time, as shown in the graphs of Figure 3.

In Table 1, LC50 and LT50 values calculated using the Spearman-Karber method are reported for both TGA- and GSH-capped QDs. TGA-QDs are characterized by lower values of both LC50 and LT50 compared to GSH-QDs, indicating a more toxic effect played by the thioglycolic acid surface compared to glutathione capping. 

To fully characterize the toxicological impact of CdTe QDs on *Hydra*, two further approaches were followed. The first one is based on the capacity of *Hydra* to regenerate missing parts of the body after amputation. During head regeneration, the development of new tentacles can be monitored by stereomicroscopy, and tentacles numbers and lengths can be scored daily to assess the potential effects played by a toxicant on this controlled process. We used a quantitative method to assay the effect of QD treatment on *Hydra* regeneration [16], calculating every day for each condition the tentacle regeneration index (TRI), which indicates the average tentacle length/*Hydra* (relative to the maximum tentacle length, assumed as 1 when the process is completed). 

**Figure 4:**
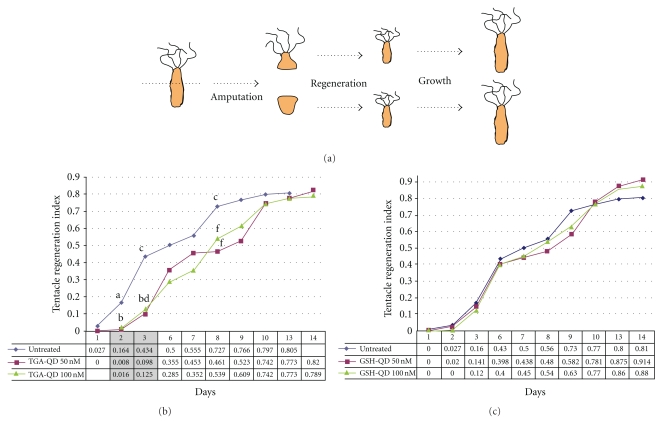
Mean tentacle regeneration indices (TRI) plotted against corresponding days of regeneration. In (a), a scheme of the regeneration is illustrated. Groups of 4 *Hydra* treated with TGA-QD (b) or GSH-QD (c) were bisected and allowed to regenerate for 14 days. For each type of QD, two different concentrations were tested (50 nM, purple line, and 100 nM, green line) and compared to untreated regenerating *Hydra*. Mean TRI values with diverse letter are significantly different (unpaired *t*-test (*P* < .001)).

As shown by the graph of Figure 4, TRI values for TGA-QD-treated animals were significantly lower compared to TRI of untreated animals. These differences were more evident during the first days of tentacle regeneration (gray shaded in the left panel of Figure 4) and less evident during the late stages of tentacle development. GSH-QD-treated animals, by contrast, were characterized by TRI similar to untreated animals, indicating for this QD type the absence of toxic effect on *Hydra* regeneration. 

**Figure 5:**
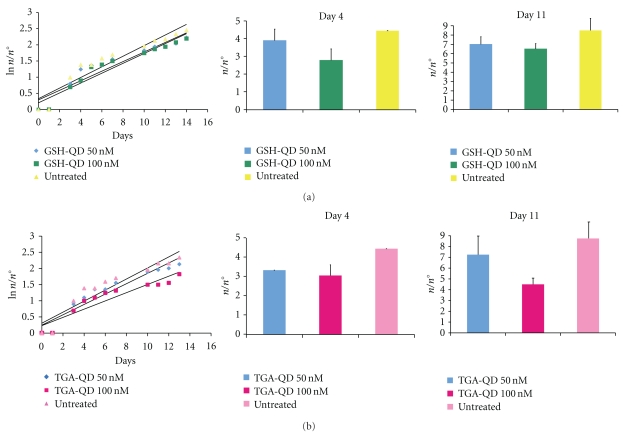
Influence of the QD treatment on *Hydra* population growth rate. Population growth test started with a population of four full-grown *Hydra*, incubated 2 h with the indicated QD, washed out, and monitored every day for bud detachment. *Hydra* were treated with GSH-QDs, dark and light green bars, (a), or TGA-QDs, dark and light pink bars, (b), washed, and equilibrated in culture solution or not treated (blue bars). The individuals were inspected daily and counted under a stereomicroscope. The logarithmic growth rate constant (*k*) is the slope of the regression line using the standard equation of logarithmic growth: ln (*n*/*n*_0_) = *kt*. Significant differences between growth rates of *Hydra* population treated with TGA-QD or untreated were obtained comparing linear regression slopes using ANOVA two-way test (*P* < .005). On the right panel, *n*/*n*° ratio (±SD) extrapolated from growth curves at days 4 and 11 is indicated.

Finally, the potential long-term toxic effects induced by CdTe QDs on *Hydra* reproductive capabilities were assayed. Growth rate of *Hydra* tissue is normally regulated by a balance between epithelial cell cycle length, phagocytosis of ectodermal cell in “excess,” and bud formation [18]. Thus, the population growth rate is an indirect measure of the *Hydra* tissue growth rate and cell viability. The growth rates of QD-treated polyps were calculated and compared to untreated animals, under regular feeding regime. As shown in the graph of Figure 5(a), the growth rate of polyps treated with GSH-QDs (two different sublethal concentrations were used) was similar to untreated animals, indicating the absence of toxic effects. Slight differences were observed only at the beginning of the experiment, as shown by the ratio *n*/*n*° (number of individuals/number of the founders) at day 4, but not later, that is, at day 11, when the differences were not significant. 

Constant growth rates of *Hydra* treated with TGA-QDs (Figure 5(b)), on the opposite, were significantly different from untreated *Hydra*. Differences in the ratio  *n*/*n*° were found all along the period of investigation, indicating an adverse effect displayed by this type of QD on *Hydra* reproductive capability.

## 4. Discussion

Despite the abundant data accumulated on the toxicity of CdTe QD on cell culture systems [20–23], it is a priority of the scientific community to assess toxicological effects at the level of whole animal. *Hydra vulgaris* represents an amenable system to study the impact of the new nanomaterials on living organism, as it is very simple; it is structured in only two cell layers, thus it can be compared to a living tissue, but it presents the complex physiology and behaviour of evolved animals. The transparency of the epithelia makes it possible to track fluorescent nanoparticles, while its sensitivity to metals makes it an ideal model for nanotoxicology studies. In this study, we investigated the effect of CdTe QDs on *Hydra*, using three different approaches, that is, assessing the effect on the polyps morphology and regenerating and reproductive capabilities. We quantitatively estimated these effects, calculating LC50 and LT50 values, tentacle regeneration index, and population growth rate, respectively, for each approach. Overall our data show that TGA-capped QDs display toxic effect compared to GSH-capped QD or to untreated animals. As by fluorescence microscopy, we were unable to evaluate the uptake of the fluorescent QDs into *Hydra* cells; at this stage, we cannot assess whether the toxicity is due to an intracellular or extracellular action played by the TGA-QDs. As we have previously shown that the positive surface charge is the crucial factor for nanoparticle internalization into *Hydra* cells [4], the observed toxicity of TGA-QD might be due to an extracellular activity, that is, binding and competing to divalent ions for membrane receptors, and we are currently investigating in to this aspect. 

Several studies suggested that the cytotoxic effects of (QDs) may be mediated by cadmium ions (Cd^2+^) released from the QD cores [24], and indeed *Hydra* has been shown sensitive to free Cd ions [10]. However, we performed similar bioassays using the supernatant of pelleted QD preparation, and we could not detect any induced toxicity, suggesting a potential role of the Cd^2+^ ions coordinated by the negative groups of the capping TGA, on the QD surface, rather than a release Cd^2+^ from the QD core. Thus, the identification of *Hydra* Cd^2+^ responsive membrane proteins would shed light on the potential mechanism of CdTe-QD-induced toxicity. The dose-dependent correlation between animal viability and QD administered further supports the hypothesis that their cytotoxicity depends on the QD actions and not on other ongoing processes, opening the path to future investigations on the intriguing cellular and molecular mechanisms underlying the CdTe-QD response in *Hydra*. Confocal laser microscopy of single-cell preparation from CdTe-QDs-treated animals imaged with organelle-specific dyes might reveal lysosomal damage attributable to the presence of reactive oxygen species (ROS), which can be formed via Cd^2+^-specific cellular pathways and/or via CdTe-triggered photo-oxidative processes involving singlet oxygen or electron transfer from excited QDs to oxygen [20, 25]. Cell biology investigation tools to check for the presence of necrosis processes or for the induction of programmed cell death (apoptosis) will help to unravel the mechanism underlying CdTe QD toxicity, which would be of invaluable help to decipher the basis of semiconductor nanocrystal toxicity also in higher organisms. 

In summary, we have shown that, when CdTe QDs interact with *Hydra* cells, this interaction induces progressive changes of cell morphology, leading finally to cell and animal death. CdTe-QD-induced cytotoxicity was associated with QD exposure time and concentration and with the surface chemistry and coating of the QD. Animal exposure for 2 hr to nanomolar doses of CdTe QD induced progressive morphological alterations, which were scored up to 72 hrs when the complete death was detected. Anterior-posterior polarity, which is normally established during bud morphogenesis and regeneration, was not affected. The induced toxicity was more pronounced in case of TGA-QD exposure, rather than GSH-QD, as shown by the dose responses curves. By treating the animals with sublethal doses of QDs, both regeneration assay and population growth rate were affected by TGA- and not GSH-capped QD, suggesting either an increased subcellular stability of GSH-QDs or a protective role played by GSH against potential Cd^2+^-induced ROS productions.

As nanoparticles may enter natural waters through sewage effluent and landfill leakages and present unknown risk to aquatic species including freshwater invertebrates, we recommend that invertebrate testing is used not only to advance the level of knowledge in nanoecotoxicology but also for investigating the behaviour and bioavailability of engineered nanoparticles in the aquatic environment through standardized tests. In conclusion, we suggest that our simple model system, up to now used mainly by a niche of biologists to study developmental and regeneration processes, has great potential to inspire many scientists working in the field of nanoscience, from chemists to toxicologists demanding new models to study the impact of nanoparticles on living organisms and their environment and to investigate the molecular basis of the bio-nonbio interactions.

## Figures and Tables

**Table 1:** Comparison of Mean (SE) lethal concentration (LC50) and lethal time (LT50) for TGA- and GSH-capped QDs.

	LC50 (nM)		QD	LT50 (hr)
Time (h)	TGA-QDs	GSH-QDs	TGA-QDs 300 nM	36
24	687.04 (27.9)	—	TGA-QDs 500 nM	24
48	232.89 (31.5)	629.99 (18.8)	GSH-QDs 500 nM	63
72	153.24 (18.9)	434.29 (15.3)	GSH-QDs 1000 nM	32

## References

[1] Bruchez M, Moronne M, Gin P, Weiss S, Alivisatos AP (1998). Semiconductor nanocrystals as fluorescent biological labels. *Science*.

[2] Alivisatos P (2004). The use of nanocrystals in biological detection. *Nature Biotechnology*.

[3] Malvindi MA, Carbone L, Quarta A (2008). Rod-shaped nanocrystals elicit neuronal activity in vivo. *Small*.

[4] Tortiglione C, Quarta A, Malvindi MA, Tino A, Pellegrino T (2009). Fluorescent nanocrystals reveal regulated portals of entry into and between the cells of Hydra. *PLoS One*.

[5] Jiang W, Kim BYS, Rutka JT, Chan WCW (2008). Nanoparticle-mediated cellular response is size-dependent. *Nature Nanotechnology*.

[6] Minchin R (2008). Nanomedicine: sizing up targets with nanoparticles. *Nature Nanotechnology*.

[7] Galliot B, Miljkovic-Licina M, de Rosa R, Chera S (2006). Hydra, a niche for cell and developmental plasticity. *Seminars in Cell and Developmental Biology*.

[8] Wilby OK The Hydra regeneration assay.

[9] Tortiglione C, Quarta A, Tino A, Manna L, Cingolani R, Pellegrino T (2007). Synthesis and biological assay of GSH functionalized fluorescent quantum dots for staining Hydra vulgaris. *Bioconjugate Chemistry*.

[10] Holdway DA, Lok K, Semaan M (2001). The acute and chronic toxicity of cadmium and zinc to two hydra species. *Environmental Toxicology*.

[11] Pollino CA, Holdway DA (1999). Potential of two hydra species as standard toxicity test animals. *Ecotoxicology and Environmental Safety*.

[12] Pascoe D, Karntanut W, Müller CT (2003). Do pharmaceuticals affect freshwater invertebrates? A study with the cnidarian Hydra vulgaris. *Chemosphere*.

[13] Karntanut W, Pascoe D (2002). The toxicity of copper, cadmium and zinc to four different Hydra (Cnidaria: Hydrozoa). *Chemosphere*.

[14] Loomis WF, Lenhoff HM (1956). Growth and sexual differentiation of Hydra in mass culture. *Journal of Experimental Zoology*.

[15] Rogach AL, Franzl T, Klar TA (2007). Aqueous synthesis of thiol-capped CdTe nanocrystals: state-of-the-art. *Journal of Physical Chemistry C*.

[16] Nolfe G, Pierobon P, Piscitelli S (1987). Tentacle regeneration in Hydra: a quantitative methodological approach. *Journal of Biomedical Engineering*.

[17] Hamilton MA, Russo RC, Thurston RV (1977). Trimmed Spearman Karber method for estimating median lethal concentrations in toxicity bioassays. *Environmental Science and Technology*.

[18] Bosch TCG, David CN (1984). Growth regulation in Hydra: relationship between epithelial cell cycle length and growth rate. *Developmental Biology*.

[19] Susha AS, Javier AM, Parak WJ, Rogach AL (2006). Luminescent CdTe nanocrystals as ion probes and pH sensors in aqueous solutions. *Colloids and Surfaces A*.

[20] Khatchadourian A, Krumova K, Boridy S, An TN, Maysinger D, Cosa G (2009). Molecular imaging of lipid peroxyl radicals in living cells with a BODIPY-*α*-tocopherol adduct. *Biochemistry*.

[21] Prasad BR, Nikolskaya N, Connolly D (2010). Long-term exposure of CdTe quantum dots on PC12 cellular activity and the determination of optimum non-toxic concentrations for biological use. *Journal of Nanobiotechnology*.

[22] Lovrić J, Bazzi HS, Cuie Y, Fortin GRA, Winnik FM, Maysinger D (2005). Differences in subcellular distribution and toxicity of green and red emitting CdTe quantum dots. *Journal of Molecular Medicine*.

[23] Khatchadourian A, Maysinger D (2009). Lipid droplets: their role in nanoparticle-induced oxidative stress. *Molecular Pharmaceutics*.

[24] Kirchner C, Liedl T, Kudera S (2005). Cytotoxicity of colloidal CdSe and CdSe/ZnS nanoparticles. *Nano Letters*.

[25] Lewinski N, Colvin V, Drezek R (2008). Cytotoxicity of nanoparticles. *Small*.

